# Karyotypic variation in *Rhinophylla pumilio* Peters, 1865 and comparative analysis with representatives of two subfamilies of Phyllostomidae (Chiroptera)

**DOI:** 10.3897/CompCytogen.v6i2.1679

**Published:** 2012-05-23

**Authors:** Anderson José Baia Gomes, Cleusa Yoshiko Nagamachi, Luís Reginaldo Ribeiro Rodrigues, Solange Gomes Farias, Jorge Dores Rissino, Julio Cesar Pieczarka

**Affiliations:** 1 Laboratory of Cytogenetics, ICB. Guamá Campus, UFPA. Perimetral Avenue, sn. Belém, PA, Brazil, 66075-900; 2 Laboratory of Genetics and Biodiversity, UFOPA, Tapajós Campus. Vera Paz Street, sn, Salé district, Santarém, PA, Brazil. 68.035-150; 3 Mammals Collection, Santa Cruz State University, UESC. Ilheus-Itabuna Highway, sn. Itabuna district. Ilhéus, BA, Brazil. 45650-000

**Keywords:** Biodiversity, Amazon rainforest, Chiroptera, cytogenetics

## Abstract

The family Phyllostomidae belongs to the most abundant and diverse group of bats in the Neotropics with more morphological traits variation at the family level than any other group within mammals. In this work, we present data of chromosome banding (G, C and Ag-NOR) and Fluorescence *In Situ* Hybridization (FISH) for representatives of *Rhinophylla pumilio* Peters, 1865 collected in four states of Brazil (Amazonas, Bahia, Mato Grosso and Pará). Two karyomorphs were found in this species: 2n=34, FN=64 in populations from western Pará and Mato Grosso states and 2n=34, FN=62 from Amazonas, Bahia, and northeastern Pará and Marajó Island (northern). Difference in the Fundamental Number is determined by variation in the size of the Nucleolar Organizer Region (NOR) accompanied with heterochromatin on chromosomes of pair 16 or, alternatively, a pericentric inversion. The C-banding technique detected constitutive heterochromatin in the centromeric regions of all chromosomes and on the distal part of the long arm of pair 15 of specimens from all localities. FISH with a DNA telomeric probe did not show any interstitial sequence, and an 18S rDNA probe and silver staining revealed the presence of NOR in the long arm of the pair 15, associated with heterochromatin, and in the short arm of the pair 16 for all specimens. The intra-specific analysis using chromosome banding did not show any significant difference between the samples. The comparative analyses using G-banding have shown that nearly all chromosomes of *Rhinophylla pumilio* were conserved in the chromosome complements of *Glossophaga soricina* Pallas, 1766, *Phyllostomus hastatus* Pallas, 1767, *Phyllostomus discolor* Wagner, 1843 and *Mimon crenulatum* Geoffroy, 1801, with a single chromosomal pair unique to *Rhinophylla pumilio* (pair 15). However, two chromosomes of *Mimon crenulatum* are polymorphic for two independent pericentric inversions. The karyotype with 2n=34, NF=62 is probably the ancestral one for the other karyotypes described for *Rhinophylla pumilio*.

## Introduction

Traditionally, the subfamily Carolliinae (*sensu* Wetterer et al. 2000) encompasses two genera: *Carollia* Gray, 1838 (10 species) and *Rhinophylla* Peters, 1865 (3 species) with wide distribution throughout South America. *Rhinophylla* consists of the smallest animals in the subfamily and has three currently recognized species: *Rhinophylla pumilio* Peters, 1865 and *Rhinophylla fischerae* Carter, 1966, with distribution on the east side of Andes in South America, and *Rhinophylla alethina* Handley, 1966 with distribution on the Pacific slope and lowlands of Colombia and Ecuador (McLellan and Koopman 2007).

Cytogenetic studies in Carolliinae have shown different rates of chromosomal evolution between both genera. The genus *Carollia* has two karyomorphs: 2n=20/21 with a multiple sex chromosome system (XX/XY_1_Y_2_), observed in most species (Yonenaga et al. 1969, Pathak et al. 1973, Stock 1975, Baker 1979, Varella-Garcia et al. 1989, Pieczarka et al. 2005), and 2n=22 with simple sex chromosome system found only in *Carollia benkeithi* Solari & Baker, 2006. On the other hand, the genus *Rhinophylla* has diversified karyotypes with four karyomorphs for *Rhinophylla pumilio* (Tables 1 and 2) and two for *Rhinophylla fischerae* (Baker and Bleier 1971, Baker 1979, Baker et al. 1987,Gomes et al. 2010). No karyotype has been described for *Rhinophylla alethina*.

**Table 1.** Cytogenetic samples of *Rhinophylla pumilio* from different localities. Numbers of sites correspond to numbers of triangles on the map (Fig. 1).

**Site**	**n**	**Locality/State**	**2N/FN**	**Methods**	**Geographical coordinates**
1	1♂+1♀	Chaves, Pará	34/62	G	00°24'55.3"S; 49°58'44.1"W
1	3♀		34/62		
2	1♂	Marituba, Pará	34/62	G, C	01°16'37.5"S; 48°20'14.9"W
3	1♂	Belém, Pará	34/62	G, C, NOR, Telomere, rDNA, CMA3	01°13'29.3"S; 48°32'59.0"W
3	1♂		34/62	G, C	
4	1♂+1♀	Santa Barbara, Pará	34/62	G	01°13'57.4"S; 48°16'34.4"W
4	4♂+2♀		34/62		
5	1♀	Capanema, Pará	34/62	C	01°24'02.5"S; 48°29'02.4"W
6	1♂	Peixe-Boi, Pará	34/62	G, C	01°11'11.0"S; 47°19'28.5"W
6	1♂		34/62	G, C, rDNA, CMA3	
7	2♂+1♀	Oriximiná, Pará	34/62	G, C	01°39'03.3"S; 56°20'30.6"W
8	1♀	Faro, Pará	34/62	G, C	02°03'53.1"S; 56°37'57.4"W
9	1♂	Juruti, Pará	34/64	G, C, NOR, rDNA	02°29'38.8"S; 56°11'27.1"W
9	1♀		34/64	G, C, rDNA	
10	1♀	Itaituba, Pará	34/64		04°16'26.6"S; 55°56'47.6"W
10	1♂		34/64	G, C, rDNA, CMA3	
11	1♂+1♀	Itaituba, Pará	34/64	G, C	04°28'20.5"S; 56°17'03.7"W
12	1♂+3♀	Itacoatiara, Amazonas	34/62	G, C	02°58'49.6"S; 58°57'51.0"W
12	1♀		34/62		
13	1♂+4♀	Potriguaçú, Mato Grosso	34/64	G, C	09°51'53.7"S; 58°13'06.8"W
14	1♂	Ilhéus, Bahia	34/62	G, C, NOR	14°47'52.0"S; 39°10'15.0"W

**Table 2.** Previous cytogenetic studies on *Rhinophylla pumilio*. Numbers of sites correspond to numbers of squares on the map (Fig. 1).

**Site**	**Region**	**Geographical coordinates**	**2n/FN**	**References**
1	Suriname	05°27'00"S; 55°12'00"W	34/64	Honeycutt et al. 1980, Baker et al. 1981
2	Suriname	03°46'00"S; 56°10'00"W	34/56	Baker and Bickham 1980
3	Colombia	04°07'43"S; 69°56'37"W	36/62	Baker and Bleier 1971
4	Brazil-Bahia	14°17'29"S; 39°51'18"W	26/48	Toledo 1973

The monophyly of the subfamily Carolliinae and the sister-group relationships of *Carollia* and *Rhinophylla* have been supported by a phylogenetic analysis based on morphological data (Baker et al. 1989, Wetterer et al. 2000, Jones et al. 2002), however molecular data are in disagreement with the advanced hypotheses (Wright et al. 1999, Baker et al. 2000, 2003b). Additionally, classical cytogenetic markers failed to provide a support for the phylogenetic relationships between *Carollia* and *Rhinophylla*, since the chromosomal homeologies could not be assigned because of the reshuffled genome of *Carollia*. In contrast, *Rhinophylla* is quite comparable to other lineages and shares a lot of chromosomal characters with representatives of the subfamilies Phyllostominae, Glossophaginae, Stenodermatinae and Desmodontinae (Baker and Bickham 1980, Baker et al. 1987, 1989).

Therefore, we analyzed, through conventional cytogenetic (G-, C- banding and Ag-NOR staining) techniques and Fluorescence *In Situ* Hybridization (FISH) with rDNA and Telomere probes, two karyotypes of *Rhinophylla pumilio* and discussed the biogeographical chromosome variation by comparing karyotypes of this species with representatives of two subfamilies of Phyllostomidae (Glossophaginae and Phyllostominae).

## Material and methods

### Specimens analyzed

**Figure 1.**
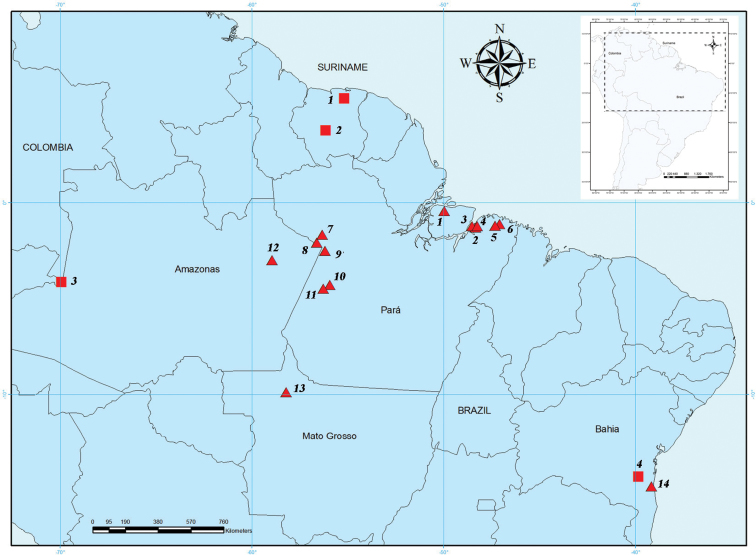
Map of collected samples of *Rhinophylla pumilio*. Squares indicate the sites from where previous cytogenetic descriptions were performed whereas triangles represent the cytogenetic samples studied herein (see Tables 1 and 2 for locality details). Numbers of sites correspond to numbers on Tables 1 and 2.

Cytogenetic preparations of *Rhinophylla pumilio* were obtained from 40 specimens collected in four states in Brazil: Pará state – 16 males and 13 females, Amazonas state – 1 male and 4 females, Mato Grosso state – 1 male and 4 females, Bahia state – 1 male (Fig. 1, Table 1). The bats were collected in the field using mist nets during the expeditions to faunal inventories. Comparative cytogenetic analyses were performed with *Glossophaga soricina* Pallas, 1766 (from Santa Barbara), *Phyllostomus hastatus* Pallas, 1767 (from Peixe-Boi), *Phyllostomus discolor* Wagner, 1843 (from Belém) and *Mimon crenulatum* Geoffroy, 1801 (from Faro). Chromosomal preparations and tissue biopsies were sent to the Cytogenetics Laboratory at Universidade Federal do Pará. Animals were fixed in 10% formalin preserved in 70% ethanol and deposited in the mammal’s collection of the Museum Paraense Emilio Goeldi, mammal’s collection of the Santa Cruz State University, Ilhéus-Bahia, Zoology Museum of the Mato Grosso Federal University and Zoology Museum of the West Pará Federal University.

### Chromosomal preparation and cell culture

The chromosome spreads were obtained from bone marrow following Baker et al. (2003a) and fibroblast primary culture following the protocols by Moratelli et al. (2002), and conventionally stained. The G-banding patterns were obtained with pepsin solution, subsequent incubation in saline solution (0,5 X SSC) at 60ºC and staining with Wright’s solution following Verma and Babu (1995). The C-banding was carried out following Sumner (1972), detection of Nucleolar Organizer Regions was performed according to Howell and Black (1980) and double staining with DAPI - CMA_3_ was performed according to Schweizer (1980).

### Fluorescence *In Situ* Hybridization (FISH)

Fluorescence *In Situ* Hybridization using digoxigenin-labeled telomeric probes (All Human Telomere Probes, Oncor) was performed according to the manufacturer’s protocol. To confirm the position of the NORs, 18S rDNA probes were amplified by BACs (Bacterial Artificial Chromosomes), labeled by nick translation and subsequently detected with avidin-Cy3 or anti-digoxigenin- FITC. Briefly, the slides were incubated in RNAse and pepsin solutions following Martins and Galetti (1998). The slides were dehydrated in ethanol series (70%, 90% and 100%), aged in a 65°C incubator for one hour, and denatured in 70% formamide/2 X SSC for one minute. The labeled probe (2 µl) was diluted in 10 µl of hybridization buffer (50% deionized formamide, 10% dextran sulfate, 0,5 M phosphate buffer 7,3 pH, 1x Denhardt’s solution), denatured at 70ºC for 15 minutes, and dropped on the slide with the denatured chromosome preparation, which was then mounted with a 24 × 24 mm coverslip. Slides then were incubated overnight at 37ºC. The hybridization signal was detected with avidin-Cy3 as described previously (Yang et al. 1995, Pieczarkaet al. 2005). The images were captured with an Axiocam Mrm CCD camera coupled on a Zeiss Axioplan 2 microscope using the Axiovision 3.0 software. The chromosomes were identified according to their morphology and inverted banding patterns using DAPI (4’,6-diamidino-2-phenylindole).

## Results

**Figure 2.**
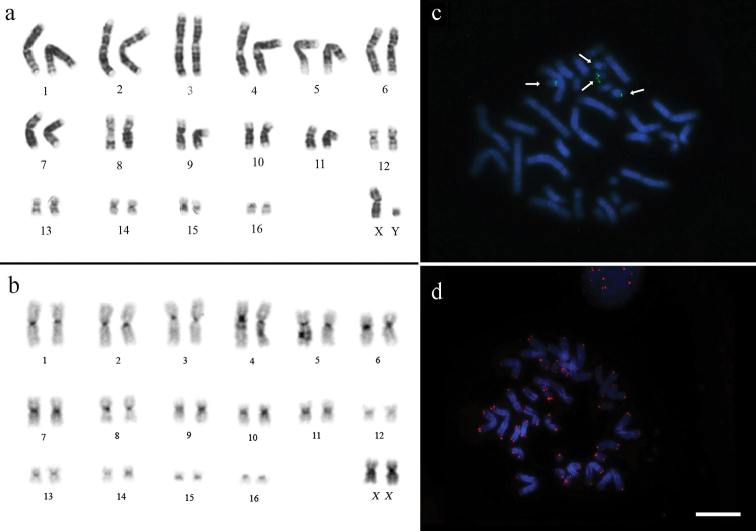
Karyotypes of *Rhinophylla pumilio* from northeastern Pará (except C-banding obtained from specimens from Amazonas state) **a** G-banding **b** C-banding **c** 18S rDNA FISH and **d** telomeric FISH. Arrows show NORs in the chromosome pairs 15 and 16. Bar = 10 µm.

**Figure 3.**
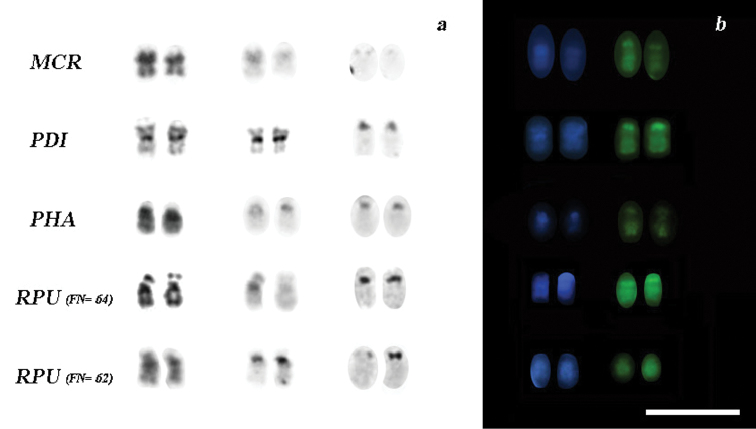
Variation of chromosome pair 15 (16 in *Rhinophylla pumilio*) in the analyzed species **a** chromosomes after G, C and Ag-NOR sequential staining **b** patterns of double staining with DAPI-CMA_3_. Bar = 10 µm.

All studied specimens of *Rhinophylla pumilio* have the same chromosome number – 2n=34. The autosomal complement consists of 15 pairs biarmed (metacentric and submetacentric) and one pair of acrocentric chromosomes (pair 16) in samples collected from Bahia, Amazonas, northeastern Pará and Marajó Island (north of Para) (Fig. 2a). In contrast, the chromosome pair 16 of specimens from west Pará and Mato Grosso is biarmed (Fig. 3a). The X chromosome is a medium-sized metacentric chromosome and the Y is a small acrocentric.

The constitutive heterochromatin was found in the centromeric regions of all chromosomes and at the distal part of the long arm of pair 15 for all specimens (Fig. 2b). Telomere sequences were observed at the tips of chromosomes (Fig. 2d). The rDNA probes and staining with silver nitrate confirmed the presence of NORs in the long arm of the pair 15 and short arm of the pair 16 (Fig. 2c). The FISH with rDNA and subsequent double staining with DAPI and CMA_3_ are in agreement with the patterns of G-bands and R-bands, respectively, where the R-bands show the tips of the chromosomes and its association with the NOR (Fig. 3b).

**Figure 4.**
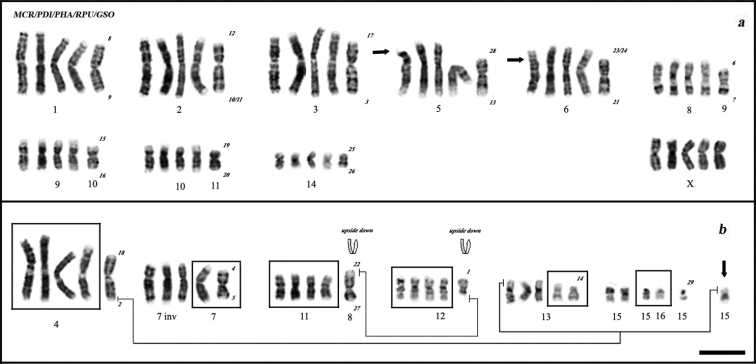
Comparative analysis using G-banded chromosomes of *Mimon crenulatum*, *Phyllostomus discolor*, *Phyllostomus hastatus*, *Rhinophylla pumilio* and *Glossophaga soricina*, from left to right **a** Conserved chromosomes among species, arrows show the centromeric position in *Mimon crenulatum* **b** Chromosomal differences among species. Black arrow indicates autapomorphic chromosome in *Rhinophylla pumilio*. Numbers (beside *Glossophaga soricina*) correspond to the chromosomal nomenclature applied to arms of *Macrotus waterhousii* in *Glossophaga soricina* according to Baker and Bass (1979). Bar = 10 µm.

The comparative analysis with *Phyllostomus hastatus*, *Phyllostomus discolor*, *Mimon crenulatum* (Phyllostominae) and *Glossophaga soricina* (Glossophaginae) (Fig. 4a) suggests that the karyotypes of *Rhinophylla pumilio* here described have nearly all chromosome pairs shared with these species, although one pair was autapomorphic to *Rhinophylla pumilio* (Fig. 4b). Analyzed species are different in the number of chromosomes (34 in *Rhinophylla pumilio* and 32 in other species) and the fundamental number (58 in *Phyllostomus hastatus*, 60 in *Mimon crenulatum*, *Phyllostomus discolor*, *Glossophaga soricina* and 62/64 in *Rhinophylla pumilio*). The heterochromatin presents in the centromeric regions of all species with additional blocks in the short and long arms of the 15th pair of *Mimon crenulatum* and *Glossophaga soricina*, respectively. Chromosomes of 5th and 6th pairs of *Mimon crenulatum* exhibit two polymorphic conditions derived probably from pericentric inversions that could cause the acrocentric and subtelocentric forms, respectively. Both specimens are heterozygous for 6th pair and homozygous for normal and rearranged forms of 5th chromosome pair. The NORs in this species are localized in the short arm of 15th pair and in the Y chromosome.

## Discussion

### Intraspecific variation in *Rhinophylla pumilio*

Our G-, C-, and Ag-NOR banding analyses have shown two distinct karyotypes for specimens of *Rhinophylla pumilio* from localities ranging more than 1000 km. The differences between these karyotypes may be caused by a pericentric inversion in the chromosome pair 16 or, alternatively, an amplification of rDNA cistrons accompanied with a faint block of heterochromatin in *Rhinophylla pumilio* with FN=64 (Fig. 3a). This segment is coincident with CMA_3_ positive staining for NOR and DAPI positive to the heterochromatic block (Fig. 3b).

Comparative analysis of karyotypes from different geographic localities (Table 2) allows discussing the morphology and number of chromosomes. Since only data of conventional staining or karyotype formula were described in the literature we had to restrict our comparisons to number and basic morphology of chromosomes. In this way, specimens of *Rhinophylla pumilio* collected on the Marajó island and northeastern Pará (Fig. 1, triangles 1, 2, 3, 4, 5, and 6) in the left side of the Amazon basin on Pará and Amazonas (triangles 7, 8 and 12) and Bahia (triangle 14) have 2n=34 and FN=62. Meanwhile, the samples from western Pará (triangles 9, 10 and 11) and Mato Grosso (triangle 13) presented the same fundamental number as specimens collected from Suriname, with 2n=34, FN=64 (Honeycutt et al. 1980, Baker et al. 1981, square 1).

Karyotype with 2n=26 and FN=48 described by Toledo (1973) (Fig. 1, Bahia, square 4) was found only in 100 km from the collection site of our sample with 2n=34 and NF=62. Varella-Garcia et al. (1989) suggested that the chromosome differences between populations of *Rhinophylla pumilio* described by Toledo (1973) and Baker and Bleier (1971) would be enough to reach the reproductive isolation between them. Nevertheless, analysis of mithocondrial DNA did not reveal sufficient genetic distance (0,3%) between two specimens from Northeastern Brazil (Pernambuco and Bahia) (Ditchfield 2000). Such distance is commonly observed within a breeding population. A re-analysis of the chromosome data from Toledo (1973) showed a disagreement with respect to the small size of the X chromosome and discordant number of chromosomes in mitotic and meiotic cells.

Another cytogenetic study on specimens of *Rhinophylla pumilio* from Colombia described a karyotype with 2n=36 and FN=62, (Baker and Bleier 1971, Fig. 1, square 3), differing from populations with 2n=34 and FN=62 probably by a chromosome fusion/fission event. Bats with karyotypes 2n=34, FN=56 (Baker and Bickham 1980, square 2) and 2n=34, FN=64 (Honeycutt et al. 1980, Baker et al. 1981, square 1) could be probably found in sympatry on the territory of Suriname.

### Intergeneric comparative analysis

Comparative analysis of chromosome banding patterns of *Rhinophylla pumilio* was undertaken with representatives of two other subfamilies of Phyllostomidae bats: *Phyllostomus hastatus*, *Phyllostomus discolor*, *Mimon crenulatum* (Phyllostominae) and *Glossophaga soricina* (Glossophaginae). Karyotypes of these species supposed to be ancestral for their respective subfamilies (Patton and Baker 1978, Baker and Bass 1979, Baker and Bickham 1980, Haiduk and Baker 1982, Baker et al. 1989) and karyotype of *Rhinophylla pumilio* with 2n=34 and FN=56 described by Baker and Bickham (1980) revealed several characters shared with the above mentioned species.

Comparative analysis revealed that there are an extensive number of conserved chromosomes shared among these species. However, *Rhinophylla pumilio* shared more characters with Phyllostominae species than *Glossophaga soricina* (Fig. 4b). Based on outgroup comparisons, Baker and Bickham (1980) proposed that the most primitive karyotype for the family Phyllostomidae is identical to that of *Macrotus waterhousii* Gray, 1843. This hypothesis together with the basal position of *Mimon waterhousii* in recent phylogenies (Baker et al. 2000, 2003b, Datzmann et al. 2010) allows to suppose the most basal nature of chromosome pairs 12 and 8q of *Glossophaga soricina* because they are homologous to the acrocentric element 22 and to short arm of the biarmed element 1/2 of *Mimon waterhousii*, respectively (in Baker and Bass 1979). However, we suggest that in the basal branch that led to peculiarity of chromosome pairs 11 and 12 of *Phyllostomus hastatus*, *Phyllostomus discolor*, *Mimon crenulatum* and *Rhinophylla pumilio*, the same chromosomes (12 and 8q of *Glossophaga soricina*) could be involved in a simple translocation from a segment on the long arm of pair 8 to short arm of the pair 12 of *Glossophaga soricina*. Alternatively, the same chromosomes would be synapomorphic in *Glossophaga soricina*, as well as in some species of the Glossophaginae subfamily, and symplesiomorphic in other species analyzed here.

Furthermore, other differences among karyotypes (Fig. 4b) are a pericentric inversion on pair 7 of *Phyllostomus hastatus* (Patton and Baker 1978) and a simple translocation involving the pairs 4 and 13 of this species as was observed by Pieczarka et al. (2005). Such events are symplesiomorphic in *Glossophaga soricina*, synapomorphic in Phyllostominae species and probably autoapomorphic in *Rhinophylla pumilio* (pair 15). Integration of data derived from multidirectional chromosome painting with chromosome probes of *Carollia brevicauda*Schinz, 1821 and *Phyllostomus hastatus* on metaphase spreads of *Glossophaga soricina* and chromosome map using probes of human chromosomes in the last species (Volleth et al. 1999) have shown that the basal position of *Glossophaga soricina* is supported by the fact that the pair 6 of human chromosomes was not disrupted. This chromosome has been assumed to be disrupted and subsequently fused with chromosome 13 of the Phyllostominae group, whereas this small segment forms an independent pair 15 in *Rhinophylla pumilio* (unpublished data).

Another interesting problem in our comparative analysis is the pair 16 in *Rhinophylla pumilio*, which has two chromosomal traits similar to those observed within representatives of genus *Phyllostomus* Lacépède, 1799. The difference between the karyotypes of *Phyllostomus hastatus* and *Phyllostomus discolor* consists of a pericentric inversion of the pair 15 (Patton and Baker 1978, Rodrigues et al. 2000). This chromosome is biarmed in *Phyllostomus discolor* and acrocentric in *Phyllostomus hastatus*, *Phyllostomus elongatus* Geoffroy, 1810, *Phyllostomus latifolius*Thomas, 1901 and *Phylloderma stenops* Peters, 1865 (Baker 1979, Baker and Bickham 1980, Honeycutt et al. 1980, Santos et al. 2002). Rodrigues et al.(2000) suggested that the biarmed state of pair 15 of *Phyllostomus discolor* could be most basal, because it has been shared with *Mimon crenulatum*, considered the most basal for the genus, and because this chromosome seems to be the result of a fusion of two acrocentric chromosomes of *Mimon waterhousii* (Patton and Baker 1978). The other species of *Phyllostomus* along with *Phyllostomus stenops* form a clade supported by the acrocentric form of the pair 15. However, the three species analyzed in this work showed different forms of the biarmed pair 15 (16 in *Rhinophylla pumilio*). The short arm of *Mimon crenulatum* represents a block of heterochromatin followed by the NOR, whereas in *Rhinophylla pumilio* the NOR appears before the heterochromatin. On the other hand, in *Glossophaga soricina* the NOR is represented at the long arm near the centromeric region accompanied by a heterochromatic block. Figure 3 shows the pattern of G- C and NOR sequential staining of pair 15 (16 in *Rhinophylla pumilio*) as well as the pattern of A/T-G/C evidenced by double staining with fluorescence DAPI and CMA_3_. The more plausible explanation is that the biarmness appeared in different branches of Phyllostomidae bats by amplification of rDNA cistrons accompanied or not with addition of heterochromatin, and possibly with other types of rearrangements.

Baker et al. (1972) defined three morphological types (submetacentric, acrocentric and subtelocentric) for the 5th chromosome pair of *Mimon crenulatum* at localities encompassing a wide geographic distance (Trinidad, Peru and Colombia). In this work, we have collected two specimens geographically apart from sites studied by Baker et al. (1972). We have found similar morphological types but G-banding analysis revealed that the acrocentric chromosome belonged to the 5th pair and the subtelocentric – to the 6th pair. That means that this polymorphism is defined by two pairs of chromosomes instead of one as it was suggested earlier.

Among species of genus *Carollia* karyotypes are highly rearranged and after the reciprocal chromosome painting Pieczarka et al. (2005) found only two chromosomes conserved *in toto* between *Carollia brevicauda* (pairs 7 and 9) and *Phyllostomus hastatus* (pairs 11 and 14). This finding suggests that they represent probably a part of the ancestral karyotype of Phyllostomidae, since they are preserved in such phylogenetically remote species. In the genus *Rhinophylla* these shared chromosomes are also presented by pairs 11 and 14 and can be also observed in others species studied herein except for the 8th pair of *Glossophaga soricina* that is partially homologous to the 11th pair of *Rhinophylla pumilio*. Therefore an analysis of the chromosomes homology among other species, especially those closely related to the genus *Carollia*, will be necessary to corroborate the sister group relationships of the genus *Carollia* and *Rhinophylla*.

Finally, we believe that variation of karyotypes along the area of *Rhinophylla pumilio* is correlated with intraspecific variation where the karyomorphs would be derived from ancestral karyotype with 2n=34, FN=62, since this karyotype is similar to other close related species at the chromosome level. However, additional analyses will be necessary to elucidate the biogeographical patterns related to the chromosome variation in *Rhinophylla pumilio*.
